# The association between elevated lipid profile and liver enzymes: a study on Bangladeshi adults

**DOI:** 10.1038/s41598-022-05766-y

**Published:** 2022-02-02

**Authors:** Rahanuma Raihanu Kathak, Abu Hasan Sumon, Noyan Hossain Molla, Mahmudul Hasan, Rakib Miah, Humaira Rashid Tuba, Ahsan Habib, Nurshad Ali

**Affiliations:** Department of Biochemistry and Molecular Biology, Shahjalal University of Science and Technology, Sylhet, 3114 Bangladesh

**Keywords:** Biomarkers, Diagnostic markers

## Abstract

Dyslipidemia, a major contributor to cardiovascular diseases, is rapidly increasing in Asian countries including Bangladesh. In addition to the cardiovascular system, abnormal lipid levels are also known to cause complications in renal and hepatic systems. The data regarding dyslipidemia and its relationship with liver enzymes are scarce for the Bangladeshi population. Therefore, this study was conducted to estimate the prevalence of dyslipidemia and determine the relationship between lipid profile and liver enzymes in Bangladeshi adults. A total of 405 participants (318 males and 87 females) were enrolled in the study. Serum levels of TG, TC, LDL, HDL and liver enzymes including ALT, AST, GGT and ALP were analyzed using standard methods. Dyslipidemia and liver function tests abnormalities were defined according to the international standard guidelines. The association between elevated lipid profile markers and liver enzyme abnormalities was assessed by logistic regression analysis. Overall, the prevalence of elevated TG, TC, LDL and low HDL were 30.9%, 23.7%, 26.2% and 78.8%, respectively. On the other hand, the prevalence of elevated liver enzymes ALT, AST, GGT and ALP were 18.8%, 21.6%, 12.9% and 21.9%, respectively. Dyslipidemia and liver enzyme abnormalities were higher in diabetic and hypertensive participants than in the healthy participants. About 61% of participants with dyslipidemia had at least one or more elevated liver enzymes. In regression analysis, an independent association was observed between serum GGT and all lipid components. In conclusion, a high prevalence of dyslipidemia and liver enzyme abnormalities were observed among the study participants. Of the four liver enzymes, the serum levels of GGT showed an independent association with all lipid components. Moreover, this study indicates that subjects with dyslipidemia often have a higher chance of having liver diseases than subjects with no dyslipidemia. However, large-scale prospective studies are needed to understand the underlying mechanisms of lipid-induced hepatic dysfunction in the Bangladeshi population.

## Introduction

Dyslipidemia is a major contributor to cardiovascular diseases (CVDs) and considered a global public health challenge^1,2^. Research over the recent decades has reported an increased prevalence of dyslipidemia among the general population^2^. Moreover, an epidemiological shift of dyslipidemia has been observed in developing nations compared to the developed nations^3^. Studies from South Asian countries have reported an upward trend of dyslipidemia among the population living in these regions^4–6^.

Dyslipidemia mediated cardiovascular diseases are the leading cause of death worldwide^7,8^. The prevalence of CVDs is rapidly increasing in the Bangladeshi population^9^. Dyslipidemias are also known to cause complications in endocrine systems, central nervous system, hepatic and renal systems^10^. Although dyslipidemia affects the major organ systems, the liver is considered to be highly affected due to its close association in lipid metabolism. The liver plays a significant role in lipid metabolism by manufacturing, storing, and transporting lipid metabolites^11^. Thus, abnormality in the lipid level, can lead to a change in liver metabolism and can damage the hepatic tissue^12^. The most common chronic liver disease represented by excess accumulation of lipids in the liver is known as non-alcoholic fatty liver disease (NAFLD). Currently, it is the most common form of hepatic disorder in the developed nations and is predicted to be identical for developing nations in the next decades^13,14^. Bangladesh is also experiencing an upward trend of fatty liver diseases, with a current prevalence of NAFLD at 33.8%^15^.

The liver enzymes aspartate aminotransferase (AST), alanine aminotransferase (ALT), alkaline phosphatase (ALP) and γ-glutamyltransferase (GGT) are generally applied as a good marker for assessing liver health^16^. Serum ALT is considered as a specific marker for hepatic damage and is mainly found in this organ^17–19^, while serum GGT is present in maximum cell surface and highly active in the kidney, liver, and pancreas^17^. Serum GGT is considered as a biomarker of alcohol consumption and hepatic injury^17^. Furthermore, GGT mediates glutathione uptake and is thought to be connected to oxidative stress and inflammation^17,20,21^. Increased levels of serum ALT and GGT are associated with various risk factors for metabolic syndrome, diabetes, and cardiovascular diseases such as obesity, hyperglycemia, dyslipidemia and increased blood pressure. Elevated levels of ALT and GGT have also been found to be associated with NAFLD^22^.

Although many studies investigated the effect of dyslipidemia on the cardiovascular system^7,23^, there are limited studies that have investigated the impact of dyslipidemia on liver functions. A study revealed that ALT and AST, the markers of hepatic dysfunction, are associated with elevated lipid profiles among the general population in the USA^24^. Despite the high occurrence of lipid-induced liver dysfunction; there are no exact statistics on the incidence of dyslipidemia and its impact on liver function in the Bangladeshi population. A few epidemiological studies reported the prevalence of dyslipidemia among the Bangladeshi adults^25–27^, but most of them were confined to a particular group rather than the more general population and did not consider some potential confounders, such as diabetes and hypertension. In addition, the association between lipid profile and liver enzymes has not been studied so far for Bangladeshi adults. Therefore, this study aims were to conduct a cross-sectional study to estimate the prevalence of dyslipidemia and evaluate the interrelationship between lipid profile and liver enzymes in Bangladeshi adults.

## Methods

### Study design and participant selection

A cross-sectional population-based study was conducted between December 2018 and February 2020 at the Department of Biochemistry and Molecular Biology, SUST, Bangladesh. In this study, a total of 405 subjects were randomly selected from general adults in the Sylhet region including academic and non-academic staff, undergraduate and graduate students. Of them, 87 were females and 318 were male subjects. Inclusion criteria: (1) both gender (18 years and above) (2) free from any severe and chronic illness and (3) willing to participate in the study. Exclusion criteria: (1) pregnant and breastfeeding women (2) participants with a present or past history of hepatotoxic drug intake, alcoholism (3) self-reported evidence of viral hepatitis and (4) subjects who did not provide blood samples or complete the questionnaire. Informed consent was obtained from all participants before their participation in the study. Ethical approval for this study was obtained from the Internal Ethics Review Board (ERB), Department of Biochemistry and Molecular Biology, School of Life Sciences, SUST (Reference number. 02/BMB/2019). All procedures of the study were carried out in accordance with relevant institutional and national guidelines and regulations.

### Anthropometric measurements

Anthropometry includes measurements of body weight, height, hip circumference (HC) and waist circumference (WC) were performed following the standard procedures described elsewhere^28–37^. Briefly, the body weight was measured by digital weighing machines (Beurer 700, Germany) to the nearest 0.1 kg^38^. The standing body height (stature) was measured to the nearest 0.1 cm using a measuring tape. BMI was calculated as the ratio of a person’s weight in kilograms (kg) divided by the height in meters squared (m^2^). The systolic (SBP) and diastolic (DBP) blood pressures were recorded by a digital BP machine (Omron, Tokyo, Japan). A mean value of two consecutive blood pressure measurements was calculated for each subject and used for the analysis.

### Sample collection and biochemical analysis

About 4 ml of fasting blood samples were collected from each participant by venipuncture using disposable needles and syringes. After centrifugation of blood samples, serum was separated and stored at − 80 °C until biochemical parameter analysis. The serum levels of fasting blood glucose (FBG), total cholesterol (TC), triglycerides (TG), low-density lipoprotein (LDL) and high-density lipoprotein (HDL) were measured by standard colorimetric methods. The concentrations of serum alanine and aspartate aminotransferase (ALT and AST), γ-glutamyltransferase (GGT), and alkaline phosphatase (ALP) were measured by kinetic methods. The available diagnostic kits were purchased from Human Diagnostic (Germany), except GGT from Vitro Scient (Egypt) to measure the biochemical parameters^38^. All the biochemical analysis was done using a semi-automated biochemistry analyzer (Humalyzer 3000, USA). All the analyses were performed according to the manufacturer’s instructions. The inter-laboratory precision and accuracy of all measurements were confirmed by regular calibration using reference standards provided in the kits.

### Diagnostic criteria

The National Cholesterol Education Program Adult Treatment Panel III (NCEP/ATP III)^39^ was followed to define dyslipidemia, as the presence of one or more measurements of TC ≥ 240 mg/dL; TG: ≥ 150 mg/dL; LDL ≥ 160 mg/dL and HDL < 40 mg/dL. Abnormal or elevated liver enzymes were confirmed if there were at least one or more measurements of ALT level > 45 U/L in men and > 34 U/L in women, AST level > 35 U/L in men and > 31 U/L in women, GGT level > 55 U/L in men and > 38 U/L in women^40^ and ALP level > 129 U/L in men and > 104 U/L in women^41^. Hypertension was defined as sustained high blood pressure (SBP ≥ 140 mm Hg and/or DBP ≥ 90 mm Hg) or the participants intake of anti-hypertensive medications. Diabetes was confirmed if the FGB ≥ 126 mg/dL, and/or with a history of usage of hypoglycemic agents and/or insulin^42^. Smoking status was defined as present smokers or non/ex-smokers. Physical activity in daily life was grouped as light (moderate housework or comfortable office jobs), moderate (swimming and walking) and heavy (jogging, lifting, carrying, or sports)^38^.

### Statistical analysis

The data were analyzed statistically using the software IBM SPSS Statistics version 23. Data are presented as means ± SD or, %. An independent sample t-test was used to compare the variables between the gender groups**.** The Chi-square test was applied to compare the prevalence of dyslipidemia and elevated liver enzymes in different groups. The relationship between lipid profile markers and liver enzymes was evaluated using multivariate logistic regression models. The *p*-value < 0.05 was considered statistically significant.

## Results

### Baseline characteristics in the sex groups

Baseline characteristics of enrolled participants are shown in Table 1. Among the 405 respondents, 318 were males and 87 were female subjects. The mean age of the total participants was 40.5 ± 12.9 years (men, 40.5 ± 12.7 years and female, 40.6 ± 13.4 years). There was a significant difference in the mean level of serum TC, TG, LDL-C, HDL-C, FBG and ALP (*p* < 0.05 for all cases) in the male and female groups. However, no significant difference was observed for BMI, WC, HC, SBP, DBP, ALT, AST and GGT in the gender groups. The prevalence of hypertension and diabetes was higher in females (46% and 49.4%, respectively) than in the male participants (41.6% and 29.9%, respectively). Regarding smoking status, about 29% of the male participants were habituated with smoking, whereas, all of the female participants were non-smokers.

**Table 1:** Baseline characteristics of the study participants.

Variables	Male (n = 318)	Female (n = 87)	Total (n = 405)	*P*-value
Age (years)	40.5 ± 12.7	40.6 ± 13.4	40.5 ± 12.9	0.959
Weight (kg)	67.1 ± 10.7	58.3 ± 9.7	65.2 ± 11.1	< 0.001
Height (cm)	165.4 ± 6.8	152.7 ± 7.9	162.7 ± 8.7	< 0.001
BMI (kg/m^2^)	24.5 ± 3.4	25.1 ± 4.1	24.6 ± 3.5	0.180
WC (cm)	86.0 ± 12.1	85.7 ± 10.9	85.9 ± 11.8	0.841
HC (cm)	92.1 ± 8.2	92.5 ± 8.9	92.2 ± 8.4	0.741
SBP (mmHg)	126.9 ± 14.5	126.2 ± 18.8	126.7 ± 15.5	0.716
DBP (mmHg)	83.6 ± 9.9	83.2 ± 10.1	83.5 ± 9.9	0.739
PP (beats/min)	76.4 ± 12.5	82.3 ± 12.1	77.7 ± 12.6	< 0.001
Glucose (mg/dL)	118.8 ± 59.4	136.8 ± 70.2	122.4 ± 63.0	0.014
TG (mg/dL)	194.6 ± 112.4	153.2 ± 94.6	185.7 ± 110.0	0.001
TC (mg/dL)	203.3 ± 73.4	228.3 ± 92.0	208.7 ± 78.3	0.021
LDL (mg/dL)	133.6 ± 62.3	162.7 ± 89.8	139.9 ± 70.0	0.001
HDL (mg/dL)	32.8 ± 13.3	35.9 ± 9.7	33.4 ± 12.6	0.016
ALT (U/L)	34.5 ± 17.2	31.7 ± 25.8	33.9 ± 19.4	0.350
AST (U/L)	27.0 ± 12.0	29.0 ± 17.1	27.4 ± 13.4	0.404
GGT (U/L)	32.8 ± 30.2	29.6 ± 28.2	32.1 ± 29.8	0.380
ALP (U/L)	100.6 ± 40.3	87.9 ± 35.0	97.6 ± 39.5	0.023
Hypertensive (%)	41.6	46.0	42.6	0.470
Diabetic (%)	29.9	49.4	34.1	0.001
Physical activity (%)				
Low	20.1	26.2	21.6	0.335
Medium	69.8	68.9	69.6	
Adequate	10.1	4.9	8.8	
Smoking status (%)				
No	70.8	100	77	< 0.001
Yes	29.2	0	23	

Data are presented as mean ± SD or %. *P*-values are obtained from independent sample t-test or Chi-square test.

### Prevalence of dyslipidemia and liver enzymes abnormalities in the sex group

**Figure 1:**
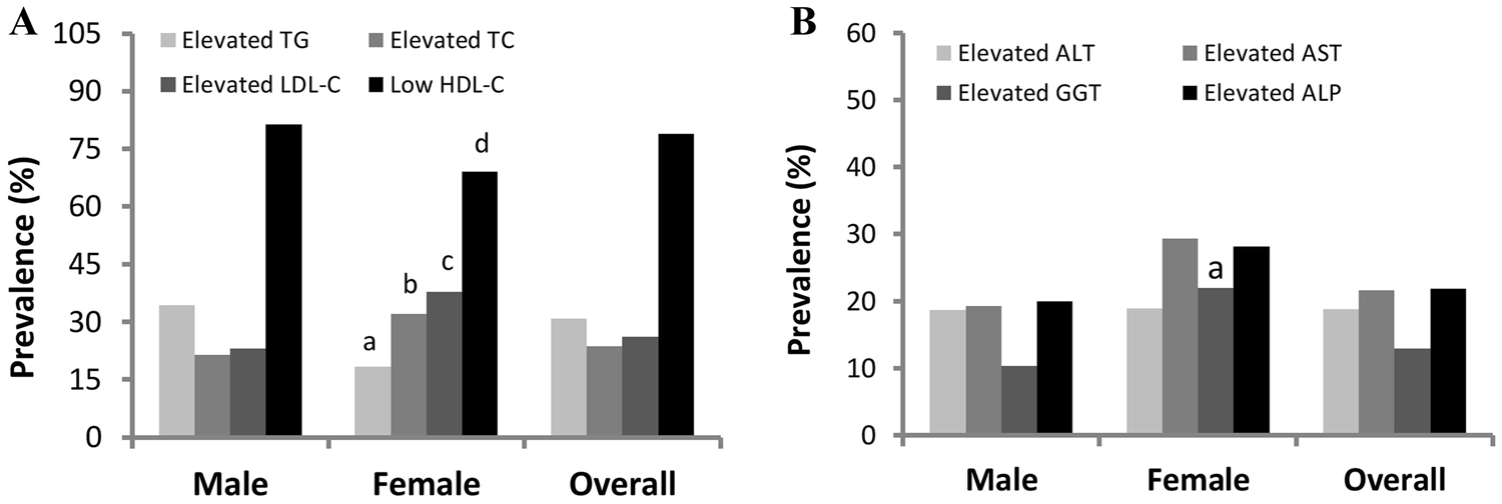
Prevalence of dyslipidemia (**A**) and elevated liver enzymes (**B**) in the gender group. In **(**A) ^a,c^*p* < 0.05; ^b,d^*p* < 0.01 when the prevalence is compared to the male group. In (**B**) ^a^*p* < 0.01 when the prevalence is compared to male group. *P*-values are obtained from Chi Square test.

The prevalence of dyslipidemia and elevated liver enzymes in the sex group is depicted in Fig. 1. Overall, the prevalence of elevated TG, TC, LDL and low HDL was 30.9%, 23.7%, 26.2% and 78.8%, respectively, in the sex group. Male participants had a high prevalence of elevated TG levels and low HDL levels, whereas, female participants had an increased prevalence of elevated TC and LDL levels. On the other hand, the overall prevalence of elevated ALT, AST, GGT and ALP were 18.8%, 21.6%, 12.9% and 21.9%, respectively. Female participants had a comparatively high prevalence of elevated liver enzymes especially AST, GGT and ALP.

### Prevalence of dyslipidemia and liver dysfunction in different groups

The prevalence of elevation and the mean level of lipid markers and liver enzymes in different health status groups are summarized in Table 2. The levels and prevalence of elevated lipid markers and hepatic enzymes were higher in hypertensive and diabetic groups compared to the healthy control group.

**Table 2:** Prevalence of elevation and levels of lipid markers and liver enzymes in various groups.

Variables	Overall (n = 405)	Healthy (n = 176)	Hypertensive (n = 172)	*P*-value^a^	Diabetic (n = 138)	*P*-value^b^
TG (mg/dL)	185.7 ± 110.0	163.6 ± 95.1	200.3 ± 116.3	0.005	211.0 ± 127.4	0.001
Elevated TG, n (%)	125 (30.9)	39 (22.3)	61 (35.5)	0.021	56 (40.6)	0.001
TC (mg/dL)	208.7 ± 78.3	183.5 ± 52.0	230.4 ± 91.5	< 0.001	235.7 ± 101.9	< 0.001
Elevated TC, n (%)	96 (23.7)	17 (9.7)	58 (33.7)	< 0.001	51 (37)	< 0.001
LDL (mg/dL)	139.9 ± 70.0	120.4 ± 46.9	157.1 ± 80.9	< 0.001	159.9 ± 94.9	< 0.001
Elevated LDL, n (%)	106 (26.2)	27 (15.3)	61 (35.5)	< 0.001	49 (35.5)	< 0.001
HDL (mg/dL)	33.4 ± 12.6	31.0 ± 9.0	35.3 ± 14.4	0.002	36.6 ± 16.4	< 0.001
Low HDL, n (%)	319 (78.8)	150 (85.2)	128 (74.4)	0.010	95 (68.8)	0.001
ALT (U/L)	33.9 ± 19.4	30.6 ± 14.5	36.6 ± 23.0	0.014	36.7 ± 22.2	0.006
Elevated ALT, n (%)	75 (18.8)	27 (15.3)	37 (22)	0.146	33 (25)	0.039
AST (U/L)	27.4 ± 13.4	23.7 ± 10.9	30.5 ± 15.7	0.003	32.2 ± 15.2	< 0.001
Elevated AST, n (%)	53 (21.6)	17 (15.2)	25 (26.3)	0.162	28 (28.9)	0.043
GGT (U/L)	32.1 ± 29.8	22.6 ± 13.2	41.0 ± 37.3	< 0.001	44.2 ± 39.8	< 0.001
Elevated GGT, n (%)	50 (12.9)	6 (3.6)	36 (21.7)	< 0.001	35 (26)	< 0.001
ALP (U/L)	97.9 ± 39.5	93.5 ± 30.5	100.3 ± 41.0	0.505	103.3 ± 47.8	0.150
Elevated ALP, n (%)	53 (21.9)	16 (14.5)	24 (25.8)	0.162	32 (33.3)	0.004

Data are presented as mean ± SD or %. *P* value^a^ is the difference between healthy and hypertensive group and *P* value^b^ is the difference between healthy and diabetic group. *P* values for mean concentrations are derived from the independent sample t-test. *P* values for prevalence (%) are obtained from Chi-square test.

### Prevalence of abnormal liver enzymes in dyslipidemic adults

About 61% of participants with dyslipidemia (having one or more elevated lipid levels) had at least one or more elevated levels of liver enzymes (Table 3). These abnormalities were significantly higher in individuals with elevated TC (except elevated ALT) and elevated TG (except elevated AST and ALP) than in individuals with normal TG and TC level (at least *p* < 0.05 for all cases). Similarly, the subjects having elevated LDL levels had significantly high levels of AST compared to the subjects having normal LDL concentrations (*p* < 0.05).

**Table 3:** Prevalence of elevated liver enzymes in dyslipidemic adults.

Variables	TG			TC			LDL			HDL		
	Normal	Elevated	*P*-value	Normal	Elevated	*P*-value	Normal	Elevated	*P*-value	Normal	Low	*P*-value
Elevated ALT	15.6	25.2	0.023	16.8	25.3	0.065	18.7	19.0	0.939	20.5	18.4	0.659
Elevated AST	20.1	25.0	0.391	16.4	32.5	0.004	17.5	30.4	0.022	20.3	22.0	0.782
Elevated GGT	9.8	19.7	0.007	9.1	24.7	< 0.001	11.5	16.5	0.197	15.4	12.2	0.455
Elevated ALP	18.5	29.7	0.051	18.0	30.7	0.027	19.2	28.0	0.124	16.4	23.5	0.259

Data are expressed as percentage (%). *P*-values are obtained from Chi-Square test.

### Association between lipid markers and liver enzymes

In all regression models, serum TG showed a significant positive association with all liver enzymes (at least *p* < 0.05 for all cases) **(**Table 4). A similar association was found for TC with ALT, AST and GGT, and for LDL with ALT and GGT (at least *p* < 0.05 for all cases). HDL showed a significant negative association only with GGT (*p* < 0.01 for model 1 and *p* < 0.05 for model 2–3).

**Table 4:** Association between lipid markers and liver enzymes.

	ALT		AST		GGT		ALP	
	OR (95% CI)	*P* for trend	OR (95% CI)	*P* for trend	OR (95% CI)	*P* for trend	OR (95% CI)	*P* for trend
**TG**								
Model 1	1.08 (1.01–1.16)	0.009	1.08 (1.01–1.13)	0.009	1.11 (1.08–1.14)	0.006	1.04 (1.01–1.07)	0.007
Model 2	1.08 (1.01–1.16)	0.024	1.08 (1.01–1.16)	0.024	1.12 (1.08–1.14)	0.021	1.04 (1.00–1.07)	0.023
Model 3	1.10 (1.22–1.38)	0.023	1.10 (1.22–1.38)	0.023	1.13 (1.03–1.24)	0.022	1.05 (1.00–1.09)	0.019
**TC**								
Model 1	1.06 (1.01–1.12)	0.007	1.07 (1.01–1.13)	0.009	1.10 (1.08–1.14)	0.006	0.98 (0.97–1.00)	0.281
Model 2	1.06 (1.00–1.12)	0.023	1.07 (1.01–1.13)	0.024	1.10 (1.08–1.14)	0.021	0.98 (0.96–1.01)	0.211
Model 3	1.07 (1.00–1.15)	0.019	1.09 (1.21–1.37)	0.023	1.11 (1.03–1.24)	0.022	0.96 (0.96–1.01)	0.211
**LDL**								
Model 1	1.04 (1.01–1.09)	0.011	1.04 (0.99–1.08)	0.096	1.04 (1.00–1.07)	0.023	0.99 (0.98–1.00)	0.279
Model 2	1.04 (1.00–1.07)	0.025	1.03 (0.98–1.08)	0.134	1.05 (1.00–1.09)	0.019	0.98 (0.97–1.01)	0.209
Model 3	1.05 (1.00–1.09)	0.023	1.05 (0.99–1.12)	0.119	1.06 (1.01–1.11)	0.014	0.99 (0.97–1.00)	0.209
**HDL**								
Model 1	0.99 (0.98–1.00)	0.279	0.94 (0.97–1.00)	0.284	− 1.04 (1.01–1.07)	0.007	− 0.99 (0.98–1.00)	0.279
Model 2	0.98 (0.97–1.01)	0.209	0.95 (0.98–1.01)	0.212	− 1.04 (1.00–1.07)	0.023	− 0.98 (0.97–1.01)	0.209
Model 3	0.99 (0.97–1.00)	0.209	0.93 (0.96–1.00)	0.212	− 1.05 (1.00–1.09)	0.019	− 0.99 (0.97–1.00)	0.209

Multivariate logistic regression was applied to evaluate the relationship between lipid profile markers and liver enzymes. Lipid profile markers were dependent variable and liver enzymes were independent variables. Three models were applied in the regression analysis. Adjusted covariates: model 1 = age, sex; model 2 = model 1 + BMI, WC and FBG; model 3 = model 2 + SBP and DBP and physical activity.

## Discussion

In the present study, a high prevalence of dyslipidemia was found among the study subjects. Of the liver enzymes, serum GGT showed an independent association with all lipid markers. To our knowledge, this is the first report on the association between lipid profile markers and liver enzymes for the Bangladeshi population.

Of the total participants, approximately 88% of subjects had at least one or more elevated lipid levels. The prevalence of dyslipidemia among Bangladeshi adults has been reported in some previous studies in the range of 20.9–75.6%^25–27,43^. The variations in the prevalence might be associated with the differences in the study population, study areas, socioeconomic status, age groups, genetic predisposition, and individual lifestyle as well as use of different cut-off values in determining dyslipidemia among the study population. Several studies suggest that abnormality in lipid profile is clinically associated with diabetes and hypertension^44,45^. Since a significant portion of our study subjects were hypertensive (42.6%) and diabetic (34.1%), which may be a possible underlying factor fueling the higher prevalence of dyslipidemia.

In the present study, the prevalence of low HDL was 78.8% which is in line with previous studies, where HDL cholesterol was the most commonly affected lipid parameter^25–27^. Some studies demonstrated that Asian people may have greater problems of low HDL cholesterol rather than other elevated lipid levels^26,46,47^.

In the sex group, a variation in the level of elevated lipids and liver enzymes was found in male and female participants. The levels of serum TG and low HDL were significantly higher in males compared to the females, whereas more female participants had an elevated TC and LDL compared to males. Industrialization and urbanization are increasing in Bangladesh at the same pace as many other countries of the world. A previous study claimed that urbanization is one of the reasons for increased dyslipidemia in Bangladesh^48^. However, in a separate analysis (data are not presented), we did not find any significant relationship between the degree of urbanization and dyslipidemia prevalence in our study subjects. Instead, we observed that dyslipidemia was more frequent among rural people; this might be for several reasons. For example, rural people are less aware of dyslipidemia and get limited health facilities compared to urban people. Poor and unhealthy diet and inadequate health knowledge among the rural people would be significant contributing factors to dyslipidemia. A more detailed investigation is needed to confirm this assumption.

The prevalence of both dyslipidemia and liver enzyme abnormalities was higher in hypertensive and diabetic groups compared to the healthy control group. These abnormalities were even more frequent among the participants who were hypertensive-diabetic. Such findings indicate that diabetes and hypertension can significantly contribute to the burden of dyslipidemia and hepatic dysfunctions. The prevalence of dyslipidemia and liver functions test markers was also found in some previous studies^44,45,49–51^. In our study, a very high predominance of elevated liver enzymes was found among participants who were dyslipidemic. This finding is partly consistent with a study in other populations, where abnormal liver function test markers were firmly identified in dyslipidemic individuals, which likely mirrored the presence of dyslipidemia in cases associated with NAFLD^52^.

In regression analysis, an independent association was found between serum GGT and all lipid components. This finding supports the claims that alterations in hepatic lipid levels can contribute to liver dysfunction. Our results and available studies suggest a crucial role of GGT in the exposition of hepatic pathology related to dyslipidemia. Liver injury in hepatic enzymes concentrations can also be caused by alcohol consumption^53^. Due to religious restriction, alcoholic drinks consumption is very uncommon in the Bangladeshi population except in rare cases. Therefore, there is less chance to have alcoholic effects on liver enzyme levels in our study subjects.

High levels of TG, TC, LDL and low HDL have been suggested to be related to NAFLD and individuals with dyslipidemia are at higher risk of developing fatty liver disease. The exact reasons for NAFLD pathogenesis are not clear yet, although it is suggested that, elevated liver function test markers are indicative of excess fat deposition in the liver and continuous assault by lipids on the liver while it exerts regular liver functions adds more workload on hepatic cells and thereby could change its physiology. Most importantly the deposited lipid particles could foster inflammation of liver tissue with the production of free radicals inside^54^. These free radicals then cause fibrosis or cell death of the liver tissue. The damaged hepatic cell can release more hepatic enzymes outside and can become more permeable because of thinning from the extra stretch due to deposited lipid remnants. Again, it has been suggested that cellular GGT may be associated with the generation of reactive oxygen species^55^, which may indicate the compromised antioxidant capacity of the liver due to the elevated lipid levels. However, the underlying mechanism of liver enzyme abnormalities in dyslipidemia remains unclear and requires further investigations.

The major strength of our study was providing information on the prevalence of abnormal lipid markers and liver enzymes panel among hypertensive and diabetic individuals. In addition, our study is the first that evaluated the relationship between lipid profile markers and liver enzymes in Bangladeshi adults. However, there are some limitations to this study. First, the cross-sectional nature of the data may affect the relationship between lipid profile markers and liver enzymes. Secondly, we considered subject’s self-reported information for a few variable measures such as level of physical activity. Moreover, the sample size was relatively small and the study was conducted in a divisional region of Bangladesh, thus our findings may not be nationally representative. However, our findings may be worth reference to future studies.

## Conclusions

A high prevalence of dyslipidemia and liver enzyme abnormalities was observed among the Bangladeshi adults; higher in diabetic and hypertensive subjects than in the healthy subjects. A higher frequency of elevated liver enzymes was observed in subjects with dyslipidemia. Of the four liver enzymes, only serum GGT showed the strongest relationship with the lipid profile markers. The elevated level of lipids and GGT in the blood may be a better indicator to monitor the progression and severity of lipid-induced hepatic dysfunctions and associated complications. This study finding indicates that dyslipidemic individuals may have a higher chance of developing liver disease than non-dyslipidemic individuals. A large-scale prospective study is needed to understand the underlying mechanisms of lipid-induced hepatic dysfunction in the Bangladeshi population.
